# *Avicennia germinans* leaf traits in degraded, restored, and natural mangrove ecosystems of Guyana

**DOI:** 10.1002/pei3.10126

**Published:** 2023-10-12

**Authors:** Sabrina Dookie, Sirpaul Jaikishun, Abdullah Adil Ansari

**Affiliations:** ^1^ Department of Biology University of Guyana Georgetown Guyana; ^2^ Faculty of Natural Sciences University of Guyana Georgetown Guyana

**Keywords:** *Avicennia germinans*, disturbances, ecosystems, Guyana, leaves, mangroves

## Abstract

Mangrove leaves have unique features that enable them to cope with shifting environmental conditions while preserving their general functionality and efficiency. We examined the morphological characteristics and chlorophyll content (spectroscopically) of 600 mature *Avicennia germinans* leaves selected from 30 trees located in one degraded, one restored, and one natural mangrove ecosystem along Guyana's coastline. Systematic sampling was carried out using the closest individual sampling method in the wet and dry seasons. We hypothesized that both habitat type and seasonality influence the leaf traits and chlorophyll content of *A. germinans.* Our findings showed that *A. germinans* leaves are mesophyllous, and traits such as leaf perimeter, area, length, width, dry mass, wet mass, turgid mass, leaf‐specific area, and relative water content showed fluctuations in ecosystems (one‐way ANOVA, *p* < .05) as well as seasonally (paired *t*‐test, *p* < .05). Substantial, positive correlations (*p* < .05, *R* > .75) were also established for over 10 leaf parameters in both seasons while PCA and multiple regression analyses further confirmed the strong relationships between leaf morphological features and their respective locations. Changes in chlorophyll concentration were most noticeable in the degraded ecosystem while variations in leaf traits were more pronounced in the restored mangrove area. This may be due to the various disturbances found in each ecosystem coupled with fluctuations in the seasons. Our results demonstrate that mangroves, to some extent, alter their plant structures to cope with environmental stressors present in the various ecosystems they thrive in to maintain their survival.

## Graphical abstract

In this study, we investigated the leaf traits of black mangrove leaves found in degraded, restored, and natural mangrove ecosystems along the coastline of Guyana, in the wet and dry seasons. Our results highlight that ecosystem type and seasonality have a significant effect on leaf traits and chlorophyll content. Correlations and regression analyses further established significant relationships between ecosystem types and leaf traits.

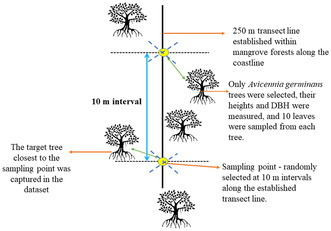

## 1 INTRODUCTION

Mangroves constitute one of the most prolific and extremely active ecosystems on Earth. (Numbere, 2018). Mangroves are well renowned for offering several significant ecological benefits and services to many nations, including Guyana. In addition to functioning as a source of nutrients and regulating the quality of water, mangroves serve as a carbon sink, protect coastlines from erosion, and sustain biodiversity by fostering food production (Dookie et al., 2022b; Nedd et al., 2021). These distinctive forests are widely known for housing a diversity of flora and fauna, reducing pollution, and producing a range of commodities including charcoal, tannins, medicine, honey, and thatch (Dookram et al., 2017; Jaikishun et al., 2017). Urbanization and sea level rise brought on by climate change, however, pose threats to these systems. Owing to overuse and the dynamic properties of the coastline, mangrove foliage has been severely depleted along portions of Guyana's coastline (Johnson‐Bhola, 2020). Additionally, the mangrove forests of Guyana are threatened by numerous natural and anthropogenic factors, which include mining, logging, farming, urban expansion, overexploitation of mangroves for manufactured products, and infrastructure construction (Winterwerp et al., 2020). Consequently, this has caused the mangrove coverage found along Guyana's coastal regions to decrease from almost 80,000 ha in 1980 to 26,115 ha in 2019 (Dookie et al., 2022a; Nedd et al., 2021).

Mangroves have evolved from land plants and developed special adaptations in their leaves, which receive and convert energy from the sun, capture carbon dioxide, release oxygen, distribute and recycle water, serve as a primary nourishment source, and drive nutritional cycles (Dawson & Goldsmith, 2018). Due to a dearth of documented studies on the topic, there currently exist knowledge gaps on the impact of mangrove habitat type and seasonality on the leaf traits of mangroves, especially in Guyana's mangrove areas which, to date, are still poorly understood. The traits of mangrove plant structures and how they may be impacted by various degrees of environmental pressures require an understanding of what is essential to the continued existence, preservation, and rehabilitation of mangrove ecosystems (Ellison et al., 2020). In this study, we have focused on the leaves of *Avicennia germinans* (*A. germinans*), which is the most dominant mangrove species occupying Guyana's coastline (Jaikishun et al., 2017). The simple, oblique leaves of this species have a length of 2–3 inches, an entire border, and an elliptical, pointed shape. They have a smooth, leathery appearance, are thick, and have a dark green upper surface and a gray‐to‐white underside (He et al., 2021; Naskar & Palit, 2014). The study of leaf characteristics is significant since traits such as leaf length, width, thickness, perimeter, area, and mass can be used to assess the current state, health, and productivity of mangroves. These measurements can be further used to provide more information on parameters such as leaf‐specific area, leaf mass per area, density, relative water content, and sclerophylly indices (Liu et al., 2017). This enables us to further comprehend the extent to which plants have developed adaptative mechanisms to manage water deficits, including stress and protoplast dehydration tolerance (Soltys‐Kalina et al., 2016). The amount of chlorophyll in the leaves is another factor that is frequently taken into account since chlorophyll content has a substantial impact on photosynthetic capacity and, as a result, plant development (Li et al., 2018). These leaf characteristics' high degree of morphological variation reflects the species' capacity to adapt to varied environmental circumstances in complex settings (Mollick et al., 2021). Since environmental circumstances are uncontrolled, such modifications may be seen as a method utilized by the plants to enhance the efficacy of water consumption and conservation, as well as light capture and shade tolerance (Khan et al., 2020; Schaepdryver et al., 2022).

Mangrove stands with reduced levels of light absorbance, leaf chlorophyll, and overall production are more susceptible to greater disturbances (Heenkenda et al., 2015). Hence, environmental pressures prompted by water availability, interstitial salinity, inundation times, variations in the frequency of climate change impacts, solar irradiance, and moisture content can influence the growth of mangrove leaves both spatially and temporally (Flores‐de‐Santiago et al., 2016). Seasonal fluctuations affect mangrove phenotypic plasticity because alterations in the pattern of precipitation can have an impact on tidal wetlands and vary the salinity of groundwater (Roth‐Nebelsick et al., 2022; Zhang et al., 2016). Also, it is anticipated that climate change will enhance seasonal variance by decreasing precipitation during dry periods (Bompy et al., 2014). During the year, there may be variations in the quantity of chlorophyll present which may be due to shifts in the biogeochemical processes of mangroves and hydrological conditions (Kumar et al., 2017; Pérez et al., 2017). In this study, we observed the severity of anthropogenic and natural disturbances in three mangrove locations along the coastline of Guyana for two seasons (dry and wet) (Table 1). Based on the extent and types of disturbances identified, ecosystems were classified as *natural ecosystems* which are mature with little to no perturbations affecting them, *restored ecosystems* that have been replanted and are presently regenerating from perturbations toward a natural state, and *degraded ecosystems* which are subjected to a significant number of disruptions from human and natural forces (Dookie et al., 2022a, 2022b). Given the aforementioned, we hypothesized that the traits and chlorophyll content of *A. germinans* leaves are influenced by ecosystem type as well as seasonality. In our perception, we believe that this study is significant since the scientific community will benefit from its findings by gaining a greater awareness of the traits of the *A. germinans* leaves found in Guyana's natural, degraded, and restored mangrove ecosystems, as well as the impact of seasonality on this specific plant structure.

**TABLE 1:** Types and extent of disturbances observed within the natural, degraded, and restored mangrove ecosystems in the wet and dry seasons.

Ecosystem type	Restored	Degraded	Natural
	Extent of disturbances		
Types of disturbances			
Natural			
Plant infestation	**	*	***
Storms/Tides	**	****	**
Erosion	**	*****	**
Insect infestation	*	*	**
Anthropogenic			
Bark stripping	*	***	*
Grazing	***	****	***
Cutting	*	***	**
Sand mining	*	**	**
Burning	*	****	**
Fishing activities	**	*****	**
Garbage dumping	*	*****	***
Infrastructure development	*	***	**
Lumbering (sawdust)	*	*****	*
Seashell mining	*	****	*

*Note*: The intensity of disturbances is denoted by: *very low, **low, ***moderate, ****high, *****very high.

## 2 MATERIALS AND METHODS

### 2.1 Description of study sites

**FIGURE 1:**
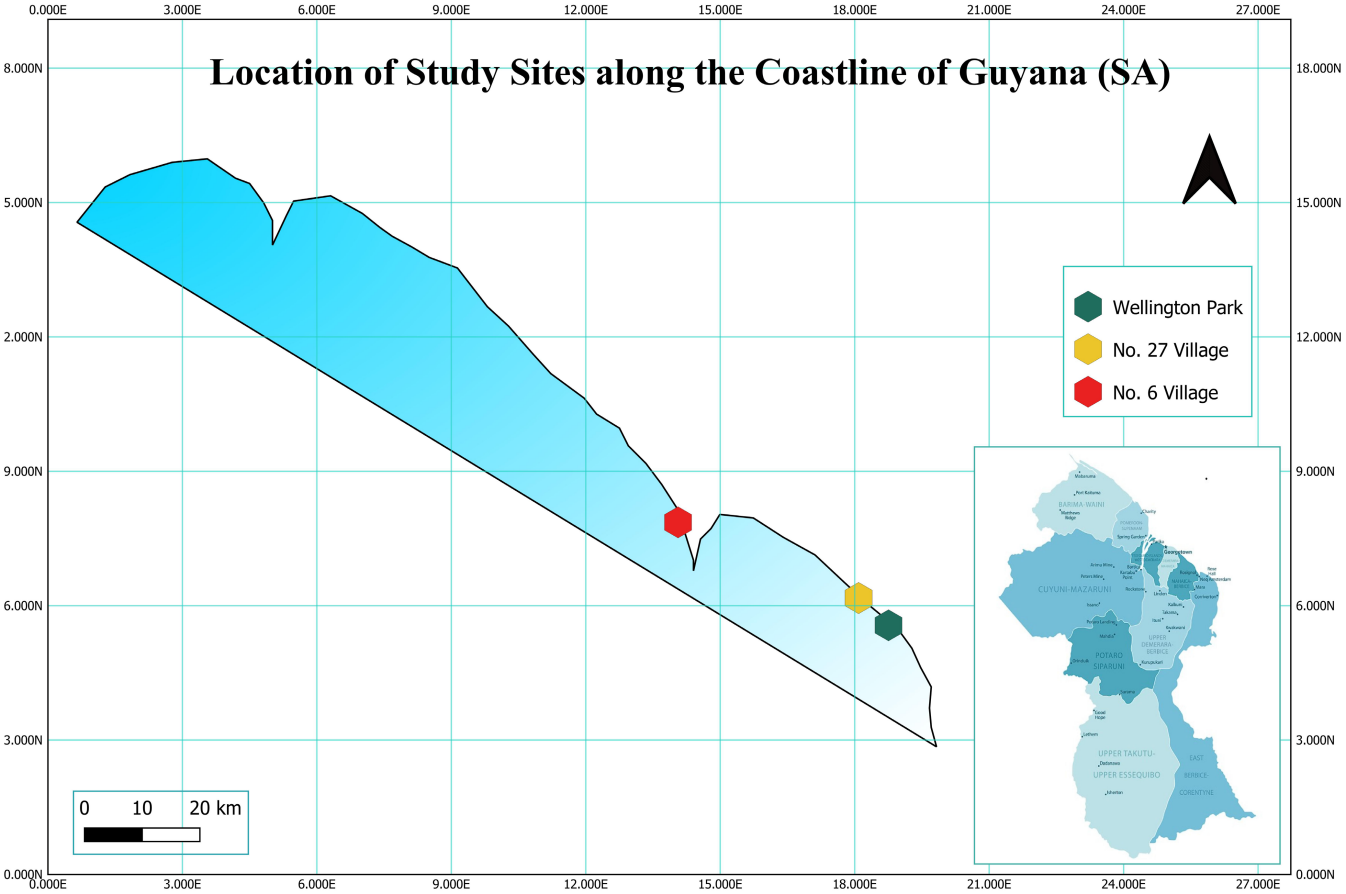
Map of the Guyana coastline showing study locations from West Coast Berbice to East Berbice Corentyne—Wellington Park (degraded ecosystem), Number 27 Village (natural ecosystem), and Number 6 Village (restored ecosystem).

Mangrove leaf sampling was conducted for two seasons in one natural (No. 27 Village—Bushlot), one degraded (Wellington Park), and one restored (No. 6 Village) mangrove ecosystem located in Regions 5 (Mahaica—Berbice) and 6 (East Berbice Corentyne) of Guyana, Northern South America (Figure 1). The restored area has juvenile mangrove trees which have not yet reached full maturity. Since 2012, this location has been artificially replanted as part of the Guyana Mangrove Restoration Project (Ministry of Agriculture, 2016). In comparison to natural and degraded ecosystems, these restored ecosystems have the greatest density of mangroves due to intensive maintenance and replanting operations. In this study, the natural mangrove system comprises extremely old, mature trees that encounter little perturbations. Although natural mangrove ecosystems may not possess very high tree densities, they are known to contribute to greater aboveground biomass (Dookie et al., 2022a, 2022b). The degraded mangrove habitat chosen (Wellington Park) was identified as one of Guyana's mangrove restoration locations, where the project's first progress was noted. However, the deterioration of the mangrove cover in this site, with its effects on water, soil composition, vegetative cover, and species, has been caused by noticeable extensive erosion driven by natural forces, improper wastewater disposal, high levels of organic matter deposition, and visible pollution in and around the forest with sawdust flowing in from the Canje Creek (Table 1). Of the three mangrove ecosystem types researched along Guyana's coastline, this type currently has the lowest tree density (Dookie et al., 2022a; Oyedotun & Hamer, 2020).

### 2.2 Experimental design

**FIGURE 2:**
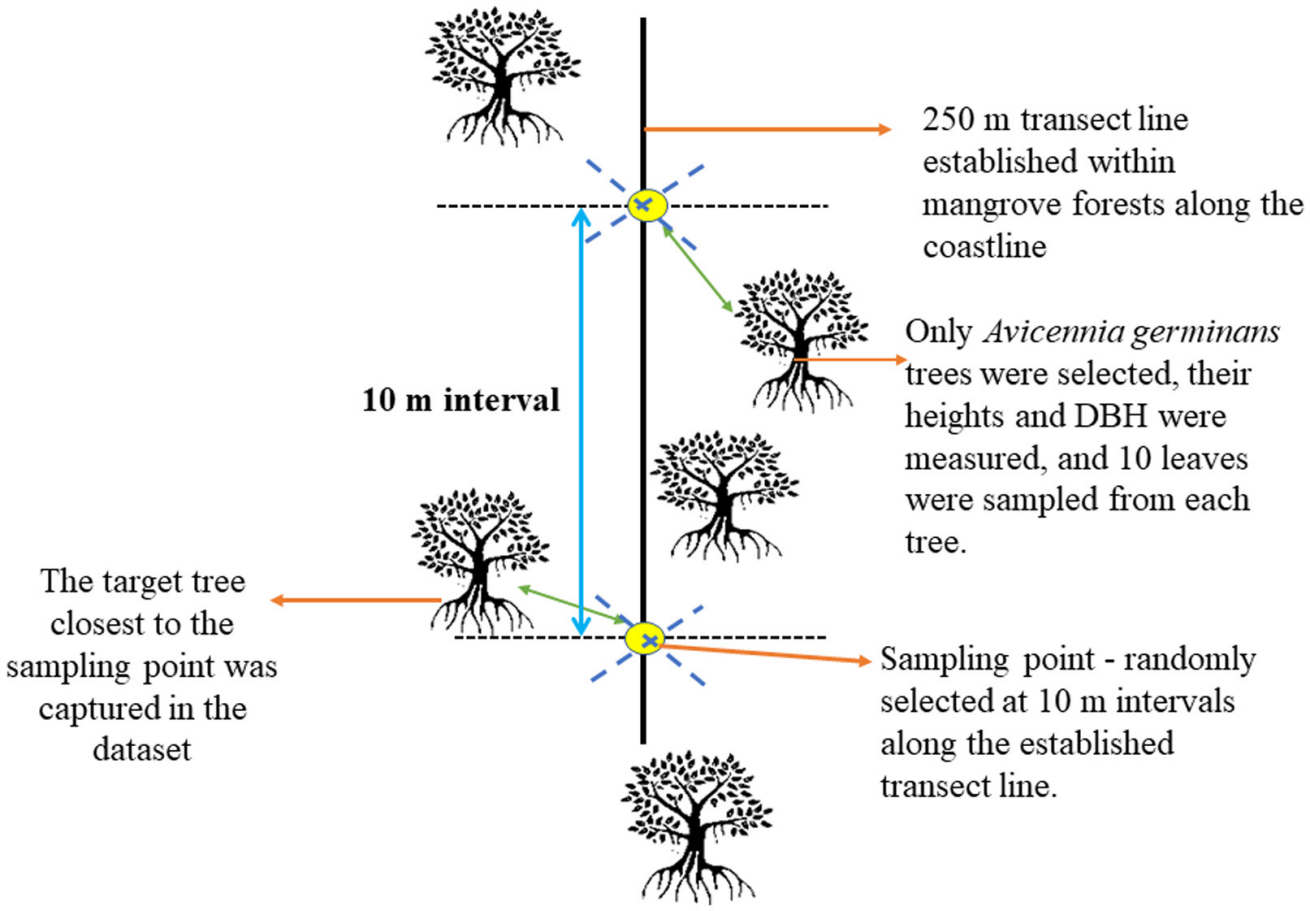
Layout of experimental design for leaf sampling using the closest individual sampling method (adapted from Elzinga et al., 1998).

The closest individual sampling method was used to sample leaves from *A. germinans* trees (Cottam & Curtis, 1956). In this plotless technique, the selection of the 10 trees was done systematically; the tree closest to the center of each sample point was selected along ten (10) random points marked within 10 m intervals along the established 250 m transect line (Figure 2). The distance between each sample location and the closest plant was then measured, the species was determined, and the plant's height and diameter at breast height (DBH) were recorded. Between the third and sixth nodes of every tree, five to seven branches that were immediately exposed to direct sunlight had 10 completely mature leaves each removed. Due to the possibility of chlorosis or damage, or to avoid selecting immature leaves, senescent and very young leaves were not selected. The green leaves were gathered, washed gently, and placed in ventilated plastic bags. Leaves collected from the restored (R), natural (N), and degraded (D) mangrove areas were then measured and described using a total of 14 parameters: length (LL), width (LW), thickness (TK), perimeter (Peri), area (AREA), fresh mass (FM), turgid mass (TM), dry mass (DM), slenderness (SLEN), leaf‐specific mass (LSM), leaf‐specific area (LSA), density (Density), Sclerophylly Index (IE), and relative water content (RWC)—in the wet season (WS) and the dry season (DS). Leaves were taken from trees with average heights ranging from 7.15 ± 0.88 m (DS)–7.16 ± 0.09 m (WS) in the degraded area, 7.55 ± 0.09 m (DS)–7.60 ± 0.09 m (WS) in the restored area, and 10.53 ± 0.17 m (DS)–10.55 ± 0.17 m (WS) in the natural area. Additionally, DBH values obtained from these trees conformed to three diameter classes: >5–10 cm, >10–20 cm, and >20–30 cm, with an average DBH of 11.13 ± 0.17 cm (DS)–11.18 ± 0.17 cm (WS) in the degraded area, 8.01 ± 0.23 cm (DS)–8.06 ± 0.23 cm (WS) in the restored area, and 20.84 ± 0.42 cm (DS)–20.86 ± 0.42 (WS) in the natural area.

### 2.3 Leaf trait measurements

The 600 leaves collected were closely examined and the following measurements were taken after standardizing instruments:

- Leaf length (LL) (cm) and leaf width (LW) (cm): were taken using a ruler on gridded paper. The longitudinal section that intersected the leaf length was used to determine the leaf length while the cross‐section that intersected the leaf length was taken as the measured leaf width.
- Leaf area (AREA) (cm^2^): was calculated using the following established allometric equation (Montgomery, 1911) is:

(1) $$A=\pi \frac{\mathrm{LL}}{2}\;\frac{\mathrm{LW}}{2}=\frac{\pi }{4}\mathrm{LL}*\mathrm{LW}$$

where LL = leaf length, LW = leaf width, and A = AREA.

The leaves of *A. germinans* are described as oval‐elliptical. Thus, the area of an elliptical leaf is:

(2) $$A=\frac{\pi }{4}$$

c Perimeter (Peri) (cm): was estimated by measuring the outline of the leaf on graph paper using a string. The string was then measured on a ruler and the perimeter was recorded.
d Thickness of the lamina (TK) (mm): was measured using a micrometer screw guage. The micrometer screw guage was placed on the leaf's central lamina area and the leaf's thickness was recorded.

(3) $$e.\;\text{Leaf slenderness}\;\left(\text{SLEN}\right)=\frac{\text{Leaf Length}\;\left(\mathrm{LL}\right)}{\text{Leaf Width}\;\left(\mathrm{LW}\right)}$$

(4) $$f.\;\text{Leaf}‐\text{specific mass}\;\left(\mathrm{LSM}\right)=\frac{\text{leaf}\;\mathrm{dry}\;\text{mass}\;\left(\mathrm{DW}\right)\;\left(g\right)}{\text{AREA}\;\left({\mathrm{cm}}^{2}\right)}$$

(5) $$g.\;\text{Specific leaf area}\;\left(\mathrm{SLA}\right)=\frac{\text{AREA}\;\left({\mathrm{cm}}^{2}\right)}{\text{leaf}\;\mathrm{dry}\;\text{mass}\;\left(\mathrm{DW}\right)\left(g\right)\;}$$

(6) $$h.\;\text{Leaf density}\;\left(\text{Density}\right)\;\left(g/{\mathrm{cm}}^{3}\right)=\mathrm{LSM}\times \frac{1}{\text{leaf thickness}\;\left(\mathrm{TK}\right)\;\left(\mathrm{cm}\right)}$$

(Hidayat & Nilawati, 2018; Witkowski & Lamont, 1991)

(7) $$i.\;\text{Sclerophylly index}\;\left(\mathrm{IE}\right)=\frac{\text{leaf}\;\mathrm{dry}\;\text{mass}\;\left(\mathrm{DW}\right)\left(g\right)}{2}\times \text{AREA}\;\left({\mathrm{dm}}^{2}\right)$$

where sclerophylly: IE > 0.6 and mesophylly: IE < 0.6 (Rizzini, 1976).

### 2.4 Leaf relative water content

The fresh weight (FW) of the leaves was determined by weighing them in the field using a digital balance immediately after they were removed from the trees. The leaves were then submersed in deionized water (diH_2_O) for 4 h after which the turgid weight (TW) of the leaves was determined by reweighing them. In order to record the dry weight (DW), samples were covered in aluminum foil and oven‐dried at 105°C for 19 min, followed by 80°C for 24 h. After achieving a consistent mass, samples were collected and their dry weight was determined by using a digital balance. The relative water content (RWC) was determined using the following formula (Pieczynski et al., 2012):

(8) $$\text{Relative Water Content}\;\left(\mathrm{RWC}\right)=\frac{\mathrm{FW}-\mathrm{DW}}{\mathrm{TW}-\mathrm{DW}}\times 10$$

### 2.5 Leaf chlorophyll content

Three leaf samples were randomly selected from each site for both seasons. Each leaf was weighed and immediately immersed in 10 mL of dimethyl sulphoxide (DMSO) in 15 mL Falcon tubes wrapped with aluminum foil. The tubes were then incubated at 65°C for 4 h. After 4 h, the samples were taken out and left to cool. Samples (3 mL) were poured into cuvettes individually with DMSO as the blank (Kumari et al., 2018). Samples were then read spectrophotometrically at 645, 663, and 470 nm. The chlorophyll content was then calculated using the Arnon method (1949) expressed in the following equations:

(9) $$\text{Chlorophyll}\;a\;\left(C_{a}\right)\;\left\{\mathrm{mg}/g\right\}\;=\left[\left(12.{\text{7A}}_{663}–2.69A_{645}\right)\;V/W\right]$$

(10) $$\text{Chlorophyll}\;b\;\left(C_{b}\right)\;\left\{\mathrm{mg}/g\right\}=\left[\left(22.{\text{9A}}_{645}–4.68A_{663}\right)\;V/W\right]$$

(11) $$\text{Total chlorophyll}\;\left(\mathrm{mg}/g\right)=C_{a}+C_{b}=\left[\left(20.{\text{2D}}_{645}+8.02D_{663}\right)\;V/W\right]$$

where W = fresh weight of tissue, A = Absorbance at specific wavelengths, and V = final volume of chlorophyll extract in dimethyl sulphoxide.

### 2.6 Data analysis

The datasets were subjected to analysis using parametric statistical tests, using a significance level of *p* < .05. The analysis was conducted using Microsoft Excel and R Studio programming software (R Studio 2023.03.1+466). The acquired values were subjected to a log10 transformation in order to increase the resemblance of the datasets to a normal distribution. The mean and standard error (S.E.) values for the 14 leaf dimensions and chlorophyll content (a, b, and total) were calculated for both the wet and dry seasons across all three ecosystems (Tables 2, 3, and 4). A one‐way analysis of variance (ANOVA) was employed to evaluate the differences in leaf traits among the three distinct types of mangrove ecosystems. Furthermore, post hoc analysis using Tukey's Honest Significant Difference (HSD) multiple comparison method was conducted to evaluate significance at a significance level of *α* = .05. Paired *t*‐tests were conducted on datasets obtained from both seasons in order to determine the statistical significance of leaf traits and chlorophyll content in each ecosystem type with respect to seasonality. Pearson correlation coefficient heat matrices were constructed in order to examine the degree of associations between leaf traits in both wet and dry seasons. Following that, multiple regression analyses were performed in order to evaluate the statistical significance of the associations between leaf traits and chlorophyll content in relation to their ecosystem type. Finally, a principal component analysis (PCA) was conducted on datasets obtained from two different seasons in order to improve the visualization of patterns and trends observed in the leaf traits of *A. germinans* across all three ecosystem types.

**TABLE 2:** Mean ± standard error (SE) of *Avicennia germinans* leaf and tree measurements taken in the dry season.

LOCATION	Tree parameters		Leaf parameters (dry season)—Mean ± SE													
	Height (m)	Diameter at breast height DBH (cm)	Length (LL) (cm)	Width (LW) (cm)	Thickness (TK) (mm)	Perimeter (Peri) (cm)	Fresh mass (FW) (g)	Turgid mass (TW) (g)	Dry mass (DW) (g)	AREA (cm^2^)	Slenderness (SLEN)	Leaf‐specific mass (LSM)	Leaf‐specific area (LSA)	Density (g/cm^3^)	Sclerophylly index (IE)	Relative water content (RWC)
Degraded area (D)	7.15 ± 0.88^c^	11.13 ± 0.17^b^	11.89 ± 0.18^b^	2.95 ± 0.05^c^	0.86 ± 0.04^b^	25.65 ± 0.39^b^	1.01 ± 0.03^b^	1.26 ± 0.03^b^	0.32 ± 0.01^b^	28.38 ± 0.74^b^	4.00 ± 0.06^a^	0.01 ± 0.01^a^	92.08 ± 1.95^b^	0.02 ± 0.01^a^	0.05 ± 0.01^a^	80.63 ± 1.20^b^
Restored area (R)	7.55±0.09^b^	8.01 ± 0.23^c^	13.73 ± 0.29^a^	4.25 ± 0.86^a^	1.24 ± 0.26^a^	29.75 ± 0.67^a^	1.39 ± 0.05^a^	1.53 ± 0.05^a^	0.28 ± 0.01^c^	46.35 ± 1.40^a^	3.32 ± 0.08^b^	0.01 ± 0.01^b^	184.77 ± 6.53^a^	0.01 ± 0.01^b^	0.07 ± 0.01^b^	87.55 ± 0.99^a^
Natural area (N)	10.53 ± 0.17^a^	20.84 ± 0.42^a^	11.56 ± 0.16^b^	3.44 ± 0.08^b^	0.78 ± 0.03^b^	25.76 ± 0.35^b^	0.92 ± 0.03^c^	1.05 ± 0.03^c^	0.36 ± 0.01^a^	31.87 ± 1.04^b^	3.53 ± 0.08^b^	0.01 ± 0.01^a^	92.41 ± 2.96^b^	0.02 ± 0.01^a^	0.06 ± 0.01^b^	75.92 ± 4.45^b^

*Note*: Letters in the table show significant pairwise associations using Tukey Honest Significant Difference (HSD) Test (*p*‐value < .05) following a one‐way ANOVA (*p*‐value <.05).

**TABLE 3:** Mean ± standard error (SE) of *Avicennia germinans* leaf and tree measurements taken in the wet season.

Location	Tree parameters		Leaf parameters (dry season)—MEAN ± SE													
	Height (m)	Diameter at breast height DBH (cm)	Length (LL) (cm)	Width (LW) (cm)	Thickness (TK) (mm)	Perimeter (Peri) (cm)	Fresh mass (FW) (g)	Turgid mass (TW) (g)	Dry mass (DW) (g)	AREA (cm^2^)	Slenderness (SLEN)	Leaf‐specific mass (LSM)	Leaf‐specific area (LSA)	Density (g/cm^3^)	Sclerophylly index (IE)	Relative water content (RWC)
Degraded area	7.16 ± 0.09^c^	11.18 ± 0.17^b^	11.95 ± 0.24^c^	3.45 ± 0.08^b^	0.84 ± 0.04^c^	26.60 ± 0.50^b^	1.21 ± 0.04^b^	1.33 ± 0.04^c^	0.35 ± 0.01^b^	33.44 ± 1.37^b^	3.54 ± 0.07^a^	0.01 ± 0.01^a^	99.50 ± 3.31^b^	0.01 ± 0.01^b^	0.06 ± 0.01^b^	87.01 ± 1.07^b^
Restored area	7.60 ± 0.09^b^	8.06 ± 0.23^c^	14.360.37^a^	4.21 ± 0.06^a^	1.11 ± 0.03^a^	32.06 ± 0.78^a^	1.69 ± 0.06^a^	1.87 ± 0.06^a^	0.44 ± 0.01^a^	48.43 ± 1.69^a^	3.41 ± 0.08^a^	0.01 ± 0.01^b^	114.31 ± 2.86^a^	3.32 ± 0.13^a^	0.11 ± 0.01^a^	88.17 ± 0.93^b^
Natural area	10.55 ± 0.17^a^	20.86 ± 0.42^a^	13.30 ± 0.18^a^	4.33 ± 0.06^a^	0.98 ± 0.03^b^	29.99 ± 0.39^a^	1.56 ± 0.04^a^	1.66 ± 0.05^b^	0.44 ± 0.01^a^	45.41 ± 1.17^a^	3.07 ± 0.05^b^	0.01 ± 0.01^ab^	105.36 ± 2.25^ab^	0.01 ± 0.01^b^	0.10 ± 0.01^a^	92.23 ± 0.57^a^

*Note*: Letters in the table show significant pairwise associations using Tukey Honest Significant Difference (HSD) Test (*p*‐value <.05) following a one‐way ANOVA (*p*‐value < .05).

**TABLE 4:** Mean ± standard error (SE) of chlorophyll content (mg/g) in *Avicennia germinans* leaves extracted from samples obtained in the wet and dry seasons.

Location	Dry season (Mean ± SE)			Wet season (Mean ± SE)		
	Chlorophyll a (mg/g)	Chlorophyll b (mg/g)	Total chlorophyll (mg/g)	Chlorophyll a (mg/g)	Chlorophyll b (mg/g)	Total chlorophyll (mg/g)
Degraded area (D)	97.33 ± 1.37	70.63^a^ ± 0.51	192.67^a^ ± 0.86	130.19 ± 1.09	189.96 ± 0.96	317.04 ± 0.83
Restored area (R)	89.15 ± 0.26	50.18^c^ ± 0.08	139.30^ab^ ± 0.25	101.02 ± 0.43	123.91 ± 0.39	224.86 ± 0.68
Natural area (N)	76.99 ± 0.19	57.46^b^ ± 0.19	81.87^b^ ± 0.17	277.93 ± 1.55	125.41 ± 0.97	234.40 ± 0.63

*Note*: Letters in the table show significant pairwise associations using Tukey Honest Significant Difference (HSD) Test (*p*‐value < .05) following a one‐way ANOVA (*p*‐value < .05).

## 3 RESULTS

### 3.1 *A. germinans* leaf trait descriptions

Average values reported for both seasons show that the restored mangrove ecosystem possessed trees with leaves that had the highest average LL [DS:13.73 ± 0.29 cm, WS: 14.36 ± 0.37 cm], LW [DS: 4.25 ± 0.86 cm, WS: 4.21 ± 0.06 cm], TK [DS:1.24 ± 0.26 cm, WS: 1.11 ± 0.03 cm], Peri [DS: 29.75 ± 0.67 cm, WS: 32.06 ± 0.78 cm], and AREA [DS: 46.35 ± 1.40 cm, WS: 48.43 ± 1.69 cm] when compared to the leaves measured in the natural and degraded mangrove areas (Tables 2 and 3). These values, however, showed fluctuations between the seasons for all mangrove sites. Furthermore, average values reported for the dry season indicated that, overall, the degraded ecosystem possessed higher values in leaf SLEN (4.00 ± 0.06), LSA (0.01 ± 0.01), Density (0.02 ± 0.01 g/cm^3^), and IE (0.05 ± 0.01) when compared to the restored and natural ecosystems. However, leaf measurements obtained within the restored ecosystem reported higher LSA values (184.77 ± 6.53) than the other two ecosystems under study. This trend, however, changed in the wet season, as marked differences were detected in the restored mangrove ecosystem, which possessed higher LSA (114.31 ± 2.86), Density (3.32 ± 0.13 g/cm^3^), and IE values (0.11 ± 0.01) when compared to the natural and degraded ecosystems. While the natural ecosystem reported average values for slenderness, LSA, LSM, Density, and IE, the degraded ecosystem reported higher SLEN (3.54 ± 0.07) and LSM values (0.01 ± 0.01) when compared to the restored and natural mangrove ecosystems. Regarding leaf masses, average values reported for the dry season (Tables 2 and 3) indicated that, overall, the restored mangrove ecosystem had the highest FW (1.39 ± 0.05 g), TW (1.53 ± 0.05 g), and RWC values (87.55 ± 0.99) when compared to the degraded and natural mangrove ecosystems. The natural mangrove ecosystem, on the other hand, had a higher DW of leaves (0.36 ± 0.01 g) than the other two types of mangrove ecosystems. A similar trend was also detected in the wet season, with the restored ecosystem exhibiting higher FW (1.69 ± 0.06 g) and TW (1.87 ± 0.06 g) when compared to the other types of mangrove ecosystems in this study. However, both restored and natural mangrove ecosystems showcased higher DW values (0.44 ± 0.01 g) when compared to the degraded area, and, unlike the restored ecosystem in the dry season, the natural ecosystem reported higher RWC values (92.23 ± 0.57) in the wet season.

### 3.2 Differences in *A. germinans* leaf traits regarding ecosystem type

#### 3.2.1 Leaf length, width, thickness, perimeter, and area

Significant differences for both seasons (one‐way ANOVA test) were reported for the mean LL [DS: (*p* = 1.38e‐08), WS: (*p* = 7.24e‐08)], LW [DS: (*p* < 2e‐16), WS: (*p* < 2e‐16)], TK [DS: (*p* < 2e‐16), WS: (*p* = 1.36e‐08)], Peri [DS: (*p* = 1.46e‐08), WS: (*p* = 8.75e‐10)], and AREA [DS: (*p* < 2e‐16), WS: (*p* = 4.21e‐14)] of leaves found within the natural, degraded, and restored mangrove ecosystems, in both wet and dry seasons. Furthermore, the Tukey HSD test showed significant differences in both seasons with respect to location in the LL [*In descending order*—DS: R > D > N, WS: R > N > D], LW [DS: R > N > D, WS: R > D > N], TK [DS: R > N > D, WS: R > N > D], Peri [DS: R > D > N, WS: R > N > D], and AREA [DS: R > N > D, WS: R > D > N].

#### 3.2.2 Leaf slenderness, leaf‐specific area, leaf‐specific mass, density, and sclerophylly indices

Significant differences in the mean values (one‐way ANOVA) were also detected for leaf SLEN (*p* = 1.14e‐09), LSM (*p* < 2e‐16), LSA (*p* < 2e‐16), Density (*p* < 2e‐16), and IE (*p* = 1.38e‐05) in the dry season with respect to location. However, in the wet season, statistically significant values reported were consistent for SLEN (*p* = 6.62e‐06), LSA (*p* = .000555), Density (*p* < 2e‐16), and IE (*p* = 1.07e‐09), but not for LSM (*p* = .0429). The Tukey HSD test showed significant differences in both seasons with respect to location in leaf slenderness (*In descending order*—SLEN [DS: D > N > R, WS: D > R > N], LSM [DS: D > N > R, WS: D > R > N], LSA [DS: R > N > D, WS: R > D > N], Density [DS: D > N > R, WS: R > D > N], and IE [DS: D > R > N, WS: R > N > D]).

#### 3.2.3 Leaf mass

Significant differences for both seasons were reported for leaf FW [DS: (*p* = 4.18e‐14), WS: (*p* = 5.97e‐12)], TW [DS: (*p* = 6.31e‐14), WS: (*p* = 1.54e‐11)], DW [DS: (*p* = 5.79e‐06), WS: (*p* = 1.63e‐06)], and RWC [DS: (*p* = 0.000546), WS: (*p* = 7.15e‐05)]. Additionally, the Tukey HSD test provided sufficient evidence to show significant differences in both seasons with respect to location in leaf FW [*in descending order*—DS: R > D > N, WS: R >N > D], TW [DS: R > D > N, WS: R > D > N], DW [DS: N > D > R, WS: R > N > D], and RWC [DS: R > D > N, WS: N > R > D].

### 3.3 Differences in *A. germinans* leaf traits regarding seasonality

#### 3.3.1 Leaf length, width, thickness, perimeter, and area

Within the natural mangrove ecosystem, statistically significant differences (using a paired *t*‐test) in seasonality were reported for LL (*p* = 1.574e‐11), LW (*p* = 2.298e‐16), TK (*p* = 5.443e‐07), Peri (*p* = 4.752e‐15), and AREA (*p* = 6.194e‐14). However, a marked decrease in significant differences was seen in the other two mangrove ecosystem types. In the degraded area, significant differences were only reported for leaf TK (*p* = 6.946e‐05) and AREA (*p* = .0195). While in the restored area, significant differences were reported only for leaf TK (*p* = .0006932) and Peri (*p* = .05481).

#### 3.3.2 Leaf slenderness, leaf‐specific area, leaf‐specific mass, density, and sclerophylly indices

Statistically significant differences (using a paired *t*‐test) in seasonality were reported in the natural mangrove ecosystem for leaf SLEN (*p* = 7.586e‐05), LSA (*p* = .000388), Density (*p* = 3.907e‐05), and IE (*p* = 4.268e‐11). Similarly, in the restored ecosystem, significant differences were reported for LSM (*p* = 4.804e‐11), LSA (*p* < 2.2e‐16), Density (*p* < 2.2e‐16), and IE values (*p* = 1.746e‐07). However, a marked decrease in significant differences was seen with respect to seasonality in the degraded ecosystem, as differences were only reported for leaf SLEN (*p* = 4.508e‐06) and IE values (*p* = .001524).

#### 3.3.3 Leaf mass

In the natural mangrove ecosystem, statistically significant differences in seasonality (paired *t*‐tests) were reported for leaf FW (*p* < 2.2e‐16), TW (*p* < 2.2e‐16), DW (*p* = 4.251e‐06), and RWC values (*p* = 6.405e‐08). Similarly, in the restored ecosystem, significant differences were reported only for FW (*p* = .0002062), TW (*p* = .0001527), and DW (*p* = 1.529e‐13). However, a marked decrease in significant differences was seen with respect to seasonality in the degraded ecosystem, as differences were only reported for leaf FW (*p* = .0156) and RWC values (*p* = .0006627).

### 3.4 Correlation among *A. germinans* leaf traits

#### 3.4.1 The restored ecosystem

**FIGURE 3:**
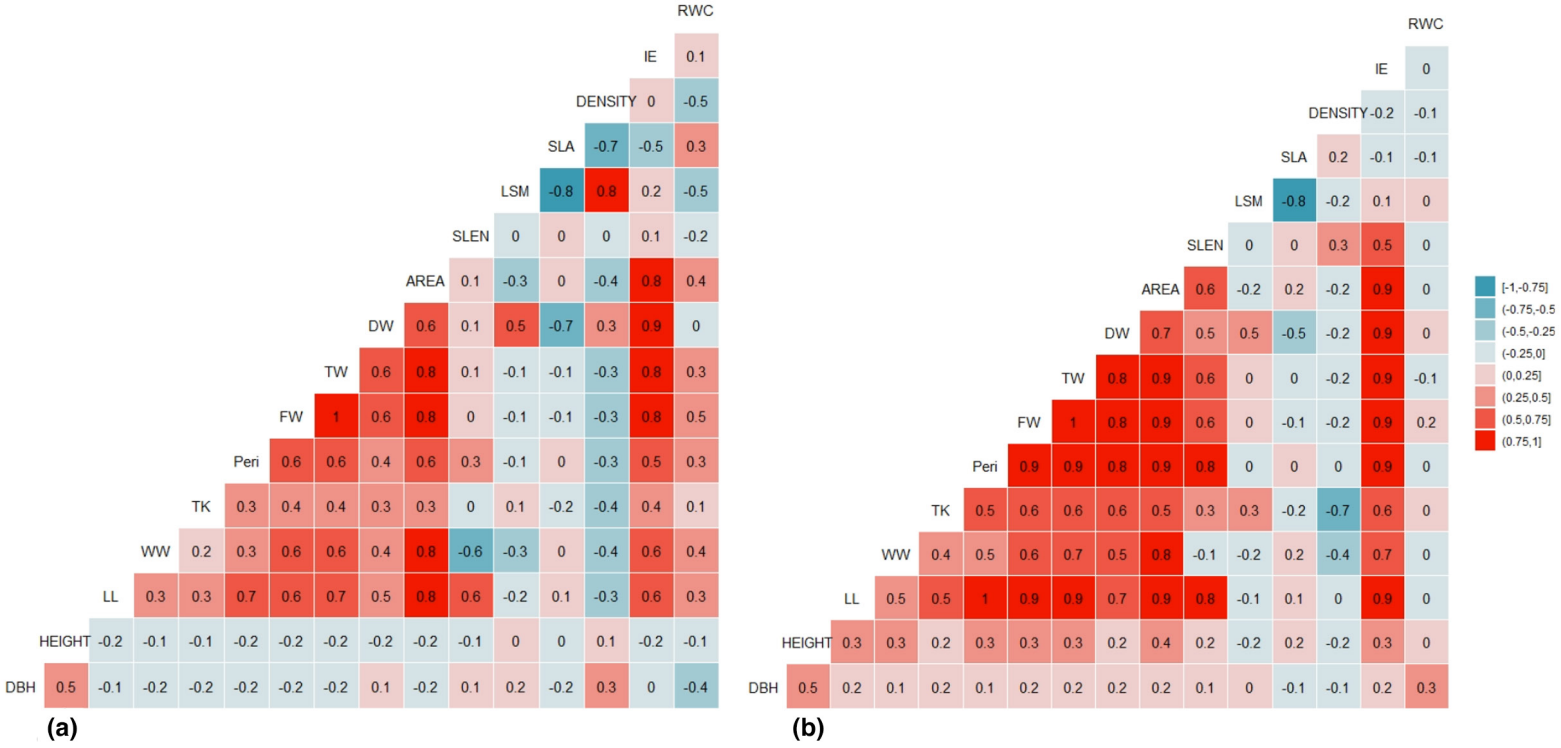
Cormatrix plots for leaf traits in the restored mangrove area in the (a) dry season, and (b) wet season.

In the dry season, strong, positive correlations (*R* ≥ .75) (Figure 3a) were detected between (Area ~ WW), (Area ~ FW), (Area ~ Peri), (Area ~ LL), (WW ~ FW), (IE ~ FW), (FW ~ TW), (IE ~ DW), (TW ~ IE), (LL ~ Peri), and (LSM ~ Density). Furthermore, strong, negative correlations (*R* ≥ −.75) were found between (LSM ~ LSA) and (Density ~ LSA). However, in the wet season, the number of positive associations between the leaf dimensions increased when compared to the dry season. Strong, positive correlations were maintained by parameters such as AREA, DW, FW, TW, and Peri. In addition, strong correlation values (*R* ≥ .75) were also detected between the following parameters: (LL ~ TW), (LL ~ FW), (LL ~ Area), (LL ~ Peri), (LL ~ DW), (LL ~ IE), (LL ~ SLEN), (Area ~ TW), (Area ~ FW), (Area ~ Peri), (Area ~ DW), (Area ~ IE), (Area ~ DW), (Area ~ WW), (Peri ~ SLEN), (Peri ~ DW), (Peri ~ IE), (Peri ~ TW), (Peri ~ FW), (DW ~ TW), (DW ~ FW), (DW ~ IE), (FW ~ IE), and (TW ~ IE). Furthermore, strong negative correlations (*R* ≥ −.75) were detected between (LSM ~ LSA) and (TK ~ Density) (Figure 3b).

#### 3.4.2 The natural ecosystem

**FIGURE 4:**
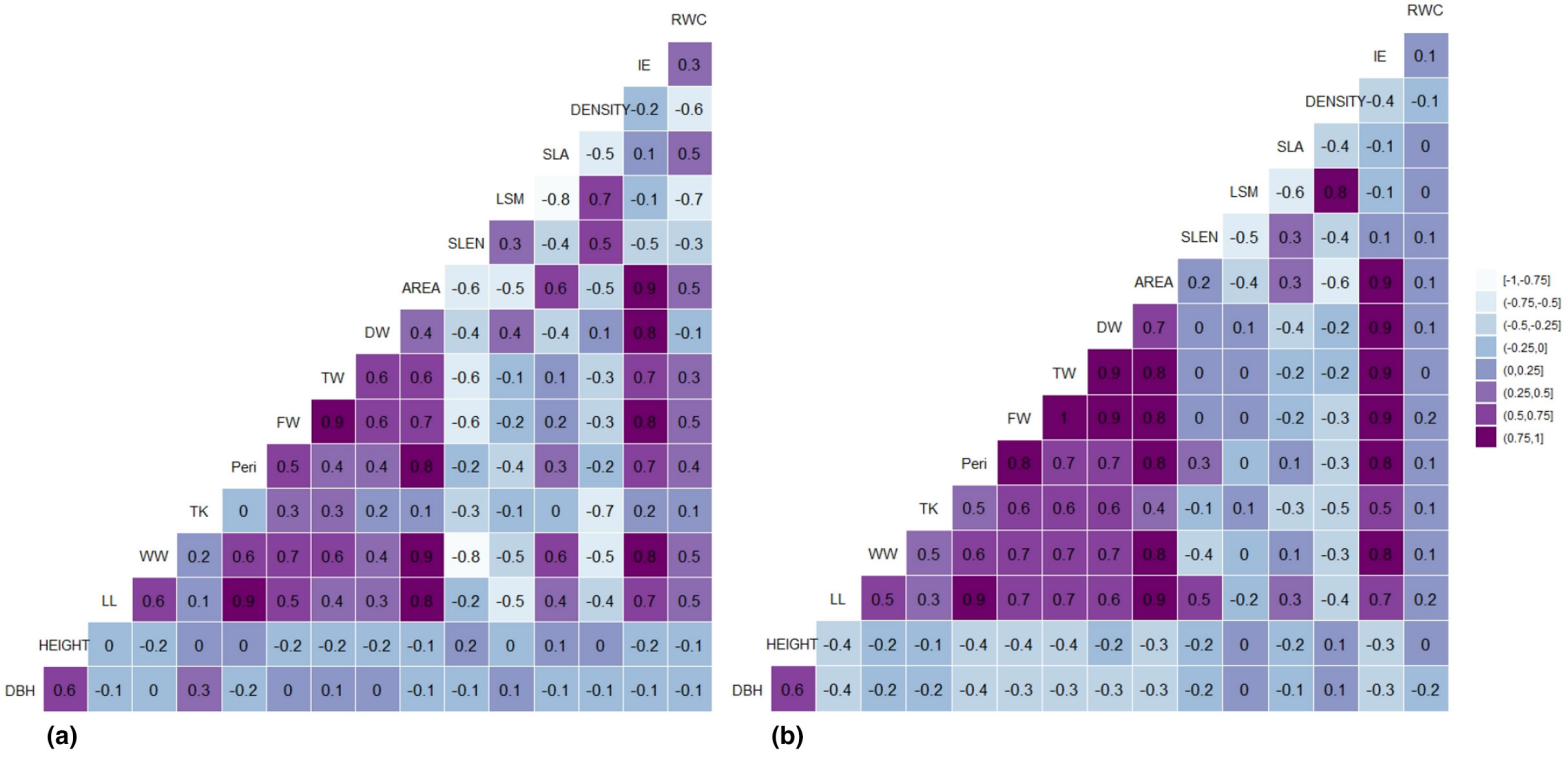
Cormatrix plots for leaf traits in the natural mangrove area in the (a) dry season, and (b) wet season.

The correlation coefficients for this ecosystem fluctuated between the wet and dry seasons; however, similar relationships were also established between parameters such as AREA, SLEN, Peri, TW, DW, and FW. Additionally, very strong relationships were established between leaf IE, AREA, and FW along with other leaf measurements. Strong positive associations (*R* ≥ .75, Figure 4a) were detected between (LSM ~ Density), (IE ~ DW), (IE ~ LL), (IE ~ Peri), (IE ~ FW), (IE ~ TW), (IE ~ WW), (IE ~ Area), (LL ~ Area), (LL ~ Peri), (Area ~ Peri), (FW ~ Area), (FW ~ WW), (FW ~ TW), and (Area ~ WW). Strong, negative correlations (*R* ≥ −0.75) were established between (SLEN ~ TW), (RWC ~ LSM), and (SLA ~ LSM). Unlike the restored ecosystem, which showcased a marked increase in the number of positive associations, the natural mangrove ecosystem maintained an adequate number of positive correlations between leaf parameters in the wet season. However, there were noticeable differences in the nature of the relationships formed, as some strong relationships were maintained by parameters such as leaf Density, IE, AREA, Peri, FW, DW, and TW, while new relationships were entirely established. Strong positive associations (*R* ≥ .75, Figure 4b) were detected between (LSM ~ Density), (IE ~ DW), (IE ~ LL), (IE ~ Peri), (IE ~ FW), (IE ~ TW), (IE ~ WW), (IE ~ Area), (LL ~ Area), (LL ~ Peri), (Area ~ Peri), (FW ~ WW), (FW ~ TW), (TW ~ WW), (FW ~ Peri), (DW ~ FW), and (DW ~ TW). Strong, negative correlations (*R* ≥ −.75, Figure 4b) were established between (Area ~ Density), (SLEN~LSM), (Area ~ LSM), and (SLA ~ LSM).

#### 3.4.3 The degraded ecosystem

**FIGURE 5:**
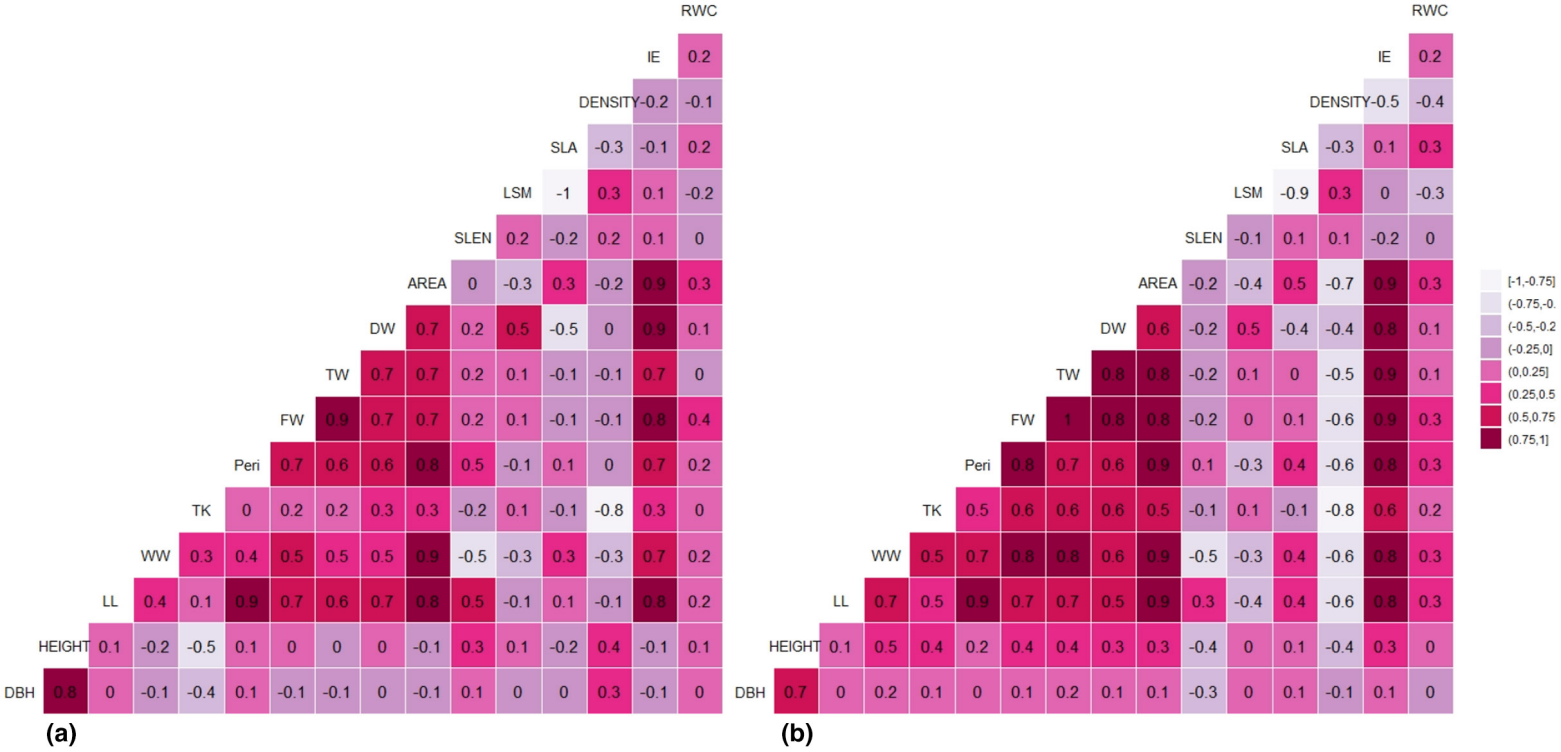
Cormatrix plots for leaf traits in the degraded mangrove area in the (a) dry season, and (b) wet season.

In contrast to what was seen with the number of positive associations in the natural and restored mangrove ecosystems, the number of positive associations in the degraded mangrove ecosystem decreased significantly in the dry season. Strong relationships between parameters such as LL, IE, AREA, and Peri were also seen and maintained among the leaf parameters in this type of ecosystem. Strong positive associations (*R* ≥ .75, Figure 5a) were detected between (Height ~ DBH), (IE ~ DW), (IE ~ LL), (IE ~ Peri), (IE ~ FW), (IE ~ TW), (IE ~ WW), (IE ~ Area), (LL ~ Area), (LL ~ Peri), (Area ~ Peri), (FW ~ TW), (Area ~ WW), and (Area ~ FW) while strong, negative correlations (*R* ≥ −.75, Figure 5a) were established between (TK ~ Density) and (SLA ~ LSM). In the wet season, the number of positive associations increased significantly between the leaf dimensions when compared to the dry season (Figure 5b). Strong, positive correlations were maintained by parameters such as AREA, Peri, LL, FW, TW, and DW. Strong correlation values (*R* ≥ .75) were detected between the following parameters: (Height ~ DBH), (LL ~ TW), (LL ~ Area), (LL ~ Peri), (LL ~ DW), (LL ~ IE), (LL ~ WW), (Area ~ TW), (Area ~ FW), (Area ~ IE), (Peri ~ Area), (Peri ~ WW), (Peri ~ IE), (Peri ~ FW), (Peri ~ TW), (DW ~ TW), (DW ~ FW), (DW ~ IE), (FW ~ TW), (FW ~ IE), (TW ~ IE), (WW ~ Area), (WW ~ IE), (WW ~ FW), and (WW ~ TW). Furthermore, strong negative correlations (*R* ≥ −.75, Figure 5b) were maintained between (LSM ~ LSA) and (TK ~ Density), as well as between (Area ~ Density).

### 3.5 Multiple regression analysis regarding *A. germinans* leaf traits and location

In the dry season, the established model predicted that, with an adjusted *R* ^2^ value of 0.47 and an overall *p*‐value <2.2e‐16, changes in the leaf parameters of the leaves located in the restored and degraded mangrove ecosystems (when compared to the intercept) were significantly associated with their location [Pr(>|*t*|) values < .05], while changes in leaf parameters in leaves found in the natural area were not significantly associated with their location [Pr(>|*t*|) values > .05].

(12) $$\begin{array}{c} \text{Model equation}\;\left(\mathrm{DS}\right)=\left(249.220^{*}\text{Intercept}\right)+\left(-1.517^{*}\text{Location}:\text{Natural}\right)\\ \;+\left(125.023^{*}\text{Location}:\text{Restored}\right) \end{array}$$

In the wet season, the established model predicted that with an adjusted *R* ^2^ value of 0.14 and an overall *p*‐value <3.991e‐11, changes in the leaf parameters of the leaves located in the restored, degraded, and natural mangrove ecosystems (when compared to the intercept) were significantly associated with their location [Pr(>|*t*|) values < .05].

(13) $$\begin{array}{c} \text{Model equation}\;\left(\mathrm{WS}\right)=\left(269.320^{*}\text{Intercept}\right)+\left(29.141^{*}\text{Location}:\text{Natural}\right)\\ \;+\left(44.205^{*}\text{Location}:\text{Restored}\right) \end{array}$$

### 3.6 Principal component analysis (PCA): *A. germinans* leaf traits

#### 3.6.1 The dry season

**FIGURE 6:**
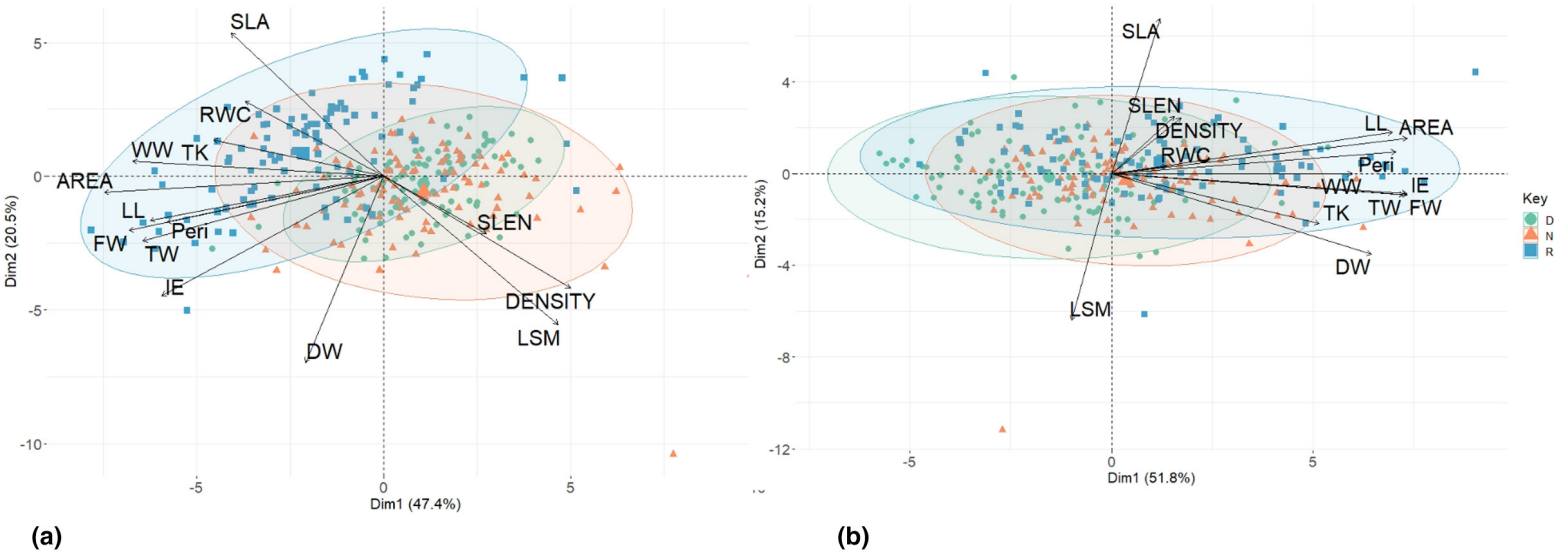
Principal Component Analysis (PCA) ordination biplots—description of leaf traits in PC1 and PC2 in the (a) dry season and (b) the wet season.

The PCA found that PC1 and PC2 explained 67.93% of the parameters' total variance. PC1 explained 47.40% of the variance and is mainly represented by the AREA, while PC2 explained 20.54% of the variance and is mainly represented by the DW of the leaves (Figure 6a). In PC1, leaf parameters of the restored ecosystem shared high values for the variables SLA, TK, WW, AREA, RWC, peri, and LL and low correlations for the variables LSM, DENSITY, DW, and SLEN. Furthermore, leaf parameters for the natural ecosystems shared high correlations for the variables IE, TW, FW, AREA, LL, WW, Peri, TK, DW, and RWC and low values for the variables DENSITY, LSM, and SLEN. However, leaf parameters in the degraded ecosystem have high correlations for the variables DENSITY, LSM, and SLEN as well as low values for the variables WW, AREA, SLA, TK, LL, FW, Peri, TW, IE, and RWC. In PC2, leaf parameters in the degraded area shared high correlations for the variables DENSITY, LSM, and SLEN as well as low values for the variables WW, AREA, SLA, TK, LL, FW, Peri, TW, IE, and RWC. Furthermore, leaf parameters in the natural mangrove area shared high correlations for the variables IE, TW, FW, AREA, LL, WW, Peri, TK, DW, and RWC and low values for the variables DENSITY, LSM, and SLEN. However, leaf parameters in the restored area showcased significant values for the variables SLA, TK, WW, AREA, RWC, Peri, and LL as well as low values for the variables LSM, DENSITY, DW, and SLEN (Figure 6a).

#### 3.6.2 The wet season

The PCA found that PC1 and PC2 explained 67.00% of the parameters' total variance. PC1 explained 51.79% of the variance and is mainly represented by the AREA and FW, while PC2 explained 15.20% of the variance and is mainly represented by the SLEN of the leaves. In PC1, leaf parameters in the restored mangrove area shared significant values for variables like AREA, IE, FW, TW, LL, Peri, WW, DW, TK, and DENSITY and low values for the variable LSM. Furthermore, leaf parameters found in the degraded area shared low values for variables like FW, DW, TW, IE, AREA, TK, Peri, WW, LL, and RWC. Within PC2, the relationships between leaf parameters found in two locations were clearly expressed. Firstly, the leaf parameters found within the restored area shared low values for variables like FW, DW, TW, IE, AREA, TK, Peri, WW, LL, and RWC. Lastly, the leaf parameters found within the natural area shared high values for the variables LSM, TK, and DW and low values for the variables SLA, DENSITY, AREA, SLEN, and LL (Figure 6b).

### 3.7 Chlorophyll content of *A. germinans* leaves

#### 3.7.1 Description of *A. germinans* leaf chlorophyll content

The average chlorophyll content values (mg/g) reported for both wet and dry seasons revealed that the leaves found within the degraded area had the highest *chlorophyll a* and *chlorophyll b*, and *total chlorophyll* content when compared to the natural and restored ecosystems (Table 4).

#### 3.7.2 Differences in *A. germinans* leaf chlorophyll content regarding ecosystem type

A one‐way ANOVA test reported insignificant differences in the mean values for *chlorophyll a* (mg/g) between locations for both seasons (*p* > .05). However, significant differences in the mean values for *chlorophyll b* (mg/g) were seen among locations for the dry season (*p* = .000169), but not for the wet season (*p* = .357). Furthermore, the Tukey HSD test revealed that within the dry season, significant differences in the mean *chlorophyll b* values were seen in the degraded area, followed by the natural area, and then the restored area (D > N > R). Additionally, significant differences in the *total chlorophyll* (mg/g) content of leaves were seen between locations for the dry season (*p* = .0105), but not for the wet season (*p* = .348). Furthermore, the Tukey HSD test revealed that within the dry season, significant variations in the mean *total chlorophyll* values differed between degraded and natural mangrove ecosystems but not the restored mangrove ecosystem (Table 4).

#### 3.7.3 Differences in *A. germinans* leaf chlorophyll content regarding seasonality

A paired *t*‐test indicated statistically significant differences in seasonality for *chlorophyll a* content in the natural mangrove ecosystem (*p* = 1.409e‐05) and the degraded mangrove ecosystem (*p* = 8.829e‐08), but not for the restored mangrove ecosystem (*p* = .1284). Furthermore, significant differences were reported for *chlorophyll b* content in the natural mangrove ecosystem (*p* = 3.77e‐15), the degraded mangrove ecosystem (*p* = 1.496e‐05), as well as the restored mangrove ecosystem (*p* = .05119). Additionally, significant differences in seasonality for *total chlorophyll* content were reported for the natural mangrove ecosystem (*p* = 1.441e‐12) and the degraded mangrove ecosystem (*p* = 9.926e‐06), but not for the restored mangrove ecosystem (*p* = .06892).

### 3.8 Multiple regression analysis: *A. germinans* leaf chlorophyll content and location

In the dry season, the established model predicted that with an adjusted *R* ^2^ value of 0.24 and an overall *p*‐value = .01385, changes in the leaf parameters of the leaves located in the natural and degraded mangrove ecosystems were significantly associated with their location [Pr(>|*t*|) values < .05], while changes in the chlorophyll content in leaves found in the restored area were not significantly associated with its location [Pr(>|*t*|) values > .05].

(14) $$\begin{array}{c} \text{Model equation}\;\left(\mathrm{DS}\right)=\left(327.84^{*}\text{Intercept}\right)+\left(-123.13^{*}\text{Location}:\text{Natural}\right)\\ \;+\left(-52.59^{*}\text{Location}:\text{Restored}\right) \end{array}$$

In the wet season, the established model predicted that with an adjusted *R* ^2^ value of 0.06 and an overall *p* value = .1803, changes in the chlorophyll content of the leaves located in the degraded mangrove ecosystems were significantly associated with location [Pr(>|*t*|) values < .05], while changes in the chlorophyll content of the leaves found in the restored and natural areas were not significantly associated with location [Pr(>|*t*|) values > .05].

(15) $$\begin{array}{c} \text{Model equation}\;\left(\mathrm{WS}\right)=\left(582.98^{*}\text{Intercept}\right)+\left(-142.39^{*}\text{Location}:\text{Natural}\right)\\ \;+\left(-191.43^{*}\text{Location}:\text{Restored}\right) \end{array}$$

## 4 DISCUSSION OF FINDINGS

### 4.1 Leaf traits of *A. germinans*

#### 4.1.1 Differences in *A. germinans* leaf traits according to ecosystem type

##### Leaf length, width, thickness, perimeter, and area

Variations in leaf origin constantly give rise to various leaf shapes and dimensions (Yu et al., 2020). Parameters such as leaf area and perimeter may be some of the most significant leaf indices for measuring how plants grow and develop in various situations since they may directly correlate to photosynthetic capacity and serve as a suitable proxy for other functional qualities like specific leaf area (SLA) (Shi, Li, Hui et al., 2020; Wright et al., 2017). In addition to being a major predictor of the growth, development, and yield production of plants, leaf geometric parameters also give crucial information for species management as well as the surveillance of disease and pests (Liu et al., 2019). Our study showed significant differences between leaf geometric parameters such as thickness, length, perimeter, width, and area with respect to location. The broader and thicker leaves present in the restored area permitted the leaves to have larger perimeters and areas when compared to the other two locations. The high degree of phenotypic plasticity in these leaf features demonstrates the species' adaptability to various environmental conditions within various locations such as sunlight, moisture, availability of nutrients, and salinity (Khan et al., 2020; Mollick et al., 2021). Plants are unable to relocate to more suitable circumstances as the environment changes. A coordinated developmental mechanism seeks to optimize light absorption while reducing the negative effects of excessive light, and when environmental temperature rises, it impacts several plant developmental features and causes morphological alterations (Li et al., 2021). The fact that certain plants prefer to grow broad leaves to optimize light collection while other plants limit their leaf size as a result of sunlight exposure being too detrimental may be one reason for the variances in leaf size seen among the diverse mangrove environments in our study (Weraduwage et al., 2015). The synchronization of phenotype, development, and environment is thought to be what allows organisms with the same genotype (in this instance, *A. germinans*) to acquire distinct phenotypes (Sultan, 2017). The leaf angle of sun‐exposed leaves varies among mangroves, which may have an impact on how species are distributed along salinity gradients. Less direct exposure due to a smaller leaf area enables the leaf to maintain photosynthesis‐friendly temperatures with a minimum amount of evapotranspiration. On the other hand, mangroves developing in high‐salinity conditions have smaller leaves (as observed in both natural and degraded mangrove locations) as another water‐saving response. Smaller leaves have greater conductivity across the leaf boundary layer, which makes it easier for the leaf temperature to acclimatize to the environment (Reef & Lovelock, 2014). The ability of roots to absorb water can be limited by salinity, which also prevents the majority of ions from entering the transpiration stream, despite the quantity of water in mangrove ecosystems. As a result, a leaf's structure, which includes its area and thickness, may also affect how much water it contains (Nguyen et al., 2017).

##### Leaf slenderness, leaf‐specific area, leaf‐specific mass, density, and sclerophylly indices

The leaf mass per area (LMA), which is comprised of density and leaf thickness, as well as its inverse, the leaf‐specific area (LSA), are each influenced by anatomical structures and chemical composition (de la Riva et al., 2016). Variations in LSM are largely due to leaf thickness, which is in turn influenced by tissue composition (Villar et al., 2013). Our study reported significant differences in leaf slenderness among the three mangrove ecosystem types, with the degraded ecosystem possessing the thinnest leaves with the highest LSM, density, and sclerophylly indices when compared to the other two locations. Leaves found within the natural and degraded areas exhibited higher LSM values when compared to the restored area, which could be attributed to factors such as species habitat, soil nutrients, light capture, and carbon loss and gain (de la Riva et al., 2017). Low LSM plants live shorter lives and exhibit higher rates of respiration and photosynthetic activity per unit leaf dry mass, while a longer lifespan and greater nutrient retention of plant material are both influenced by greater LSM (Gara et al., 2021). Such changes in LSM need the inclusion of additional plant material to attain a given leaf area for light absorption, which means a greater cost of construction per unit leaf area (Gratani et al., 2018). High LSA values, like those found in the restored region, indicate that the leaves are more functional since they devote more energy to photosynthesis than structural tissues do. Yet, despite being more resilient to resource constraints in the soil, leaves exhibiting low specific leaf area values (those seen in natural and degraded environments) are less productive (Sereneski‐Lima et al., 2022). Moreover, low leaf densities (found in both natural and restored ecosystems) may be owing to the existence of an increased amount of air spaces, which improves leaf conductance and facilitates photosynthesis. Nevertheless, large densities can come from an abundance of mesophyll or lignified materials, which may be essential for leaf rigidity and, subsequently, the survivability of the leaf and species of plants (exhibited in the degraded area) (Xiong et al., 2016). Mangroves are not considered to be a sclerophyllous ecosystem, despite the presence of thick, leathery leaves on mangrove trees. Many leaf traits of true mangroves are more similar to those found in plants from drier conditions, indicating the notion that a saline environment produces physiological sensitivity to periods of dryness (Quadros et al., 2021). The presence of sclerophylly in mangrove species might be viewed as a mechanism used by the plants to increase the effectiveness of water usage when environmental conditions are uncontrollable (Sereneski‐de Lima et al., 2013). Sclerophylly was not exhibited in the *A. germinans* leaves within this study, as all the leaves found within the three mangrove locations have IE values less than 0.6, which describes them as mesophyllous in nature according to the Rizzini Index.

##### Leaf mass

Like other vegetation, mangroves need to absorb and retain water to keep their leaves moist. However, maintaining optimum water and ion balances in a saline environment presents unique difficulties (Nguyen et al., 2017). The amount of water per unit of dry/fresh weight and unit of water at complete hydration are two ways to characterize the moisture content of plant tissue. In essence, RWC is the absolute quantity of water needed by a plant to attain total saturation artificially. This makes it a helpful indication of the condition of the water balance of a plant as the type and maturity of plant materials can have a big impact on this relationship (Fariñas et al., 2019). Mangrove leaves have a lot of water per unit of area (salt succulence), and this amount increases with salinity. High water content reduces the demand for evapotranspiration since it enhances the heat capacity of the leaf (Reef & Lovelock, 2014). Leaves in the restored and natural ecosystem had larger fresh masses, turgid masses, dry masses, and overall RWC when compared to the leaves in the degraded mangrove area. Schaepdryver et al. (2022) confirmed that similar to the results of this study, the water uptake and retention of plant leaves can vary among mangrove species that are found in various environments under different existing conditions. Several measurements of leaf size, such as leaf weight and area, were shown to have a power‐law relationship. “Diminishing returns” describes when the dry mass of a leaf increases, the leaf area eventually decreases (Wright et al., 2017). A larger surface will often enhance the interacting area to capture the light under the continued investment of biomass in a leaf, which unquestionably increases the photosynthetic capacity (Dawson & Goldsmith, 2018). Big leaves, such as those present in the natural and restored mangrove areas, can enhance the foliar surface's ability to use light, but they also require more biomass to grow larger leaves. Yet, the amount of water in the leaves is crucial to photosynthesis. The proportions of leaf dry weight to fresh weight for numerous individual leaves are not consistent (Huang et al., 2019). The amount of leaves can increase despite a reduction in mean leaf size in some mangrove plants (such as those in the degraded mangrove area). This is seen as a tree's attempt to handle increased stress and ensure greater water usage efficiency. Because there is less water available, the leaves have less capacity to store water, which results in decreased water content in leaves in highly stressed environments (Noor et al., 2015). Our results suggest that trees found within the restored and natural mangrove ecosystems are not severely stressed by environmental conditions such as intense aridity and salinity. This can be seen in their increased leaf sizes (as seen by leaf dry masses) for the uptake of more water for use in photosynthesis and transpiration.

### 4.2 Differences in *A. germinans* leaf traits according to seasonality

#### 4.2.1 Leaf length, width, thickness, perimeter, and area

Mangrove phenology is influenced by seasonal variations in temperature, precipitation, nutrient availability, and freshwater (Zhang et al., 2016). Raindrop impact on leaves is a frequent occurrence that affects numerous processes such as leaf wax erosiveness, moisture content, gas exchange, and the storage and capture of rainwater by canopies. Rainfall affects the surface area and leaf angle of inclination, causing elastic movements such as local distortion, flapping, twisting, and bending (Roth‐Nebelsick et al., 2022). This can affect the overall geometry of the leaves, causing them to exhibit marked changes during the wet season in mangrove forests. Our study revealed that seasonality has a significant effect on leaf geometric parameters in the natural area but little to no effect on leaves in restored and degraded areas. This suggests that *A. germinans* exhibits a degree of resistance to changes induced by seasonality with respect to leaf geometric parameters. However, there are a number of reasons that may encourage variability in leaf parameters in the wet and dry seasons. For instance, topsoil salinity in Caribbean mangroves and wetlands may vary both in the wet and dry seasons. Additionally, by reducing the amount of precipitation during the dry season, climate change is predicted to increase seasonal variation (Bompy et al., 2014). Increasing rates of soil evaporation would result in a rise in soil salinity if rising CO_2_ is accompanied by greater temperatures (as observed in dry periods), which will reduce root water absorption and decrease projected CO_2_‐related gains in growth (Steppe et al., 2018). Rainfall also affects mangrove ecosystems through the soil, reducing soil salinity and temperature, as well as through changing the atmosphere by raising humidity levels and lowering the temperature (Reef & Lovelock, 2014). Soil and atmospheric phenomena concerning rainfall have repeatedly been utilized to explain substantial increases in plant parts such as stems and leaves following rainfall since both elements are documented to positively enhance mangrove water replenishment and hence rehydrate live cells (Santini et al., 2014). Trees found in the natural area exhibited larger DBH values, which is an indication of broader trunks in mature trees. This, therefore, suggests that transport vessels such as xylem are abundant, thus facilitating the increased uptake and transport of water (when it becomes available in the wet season) for food production by photosynthesis. Trees found within the degraded and restored areas reported smaller DBH values, which indicated that trees were either immature (young) (restored area) or starved of soil nutrients due to excessive disturbances (degraded area) (Nguyen et al., 2020).

#### 4.2.2 Leaf slenderness, leaf‐specific area, leaf‐specific mass, density, and sclerophylly indices

Variations in leaf traits can be in response to climatic fluctuations through modifications to components such as leaf thickness, area, dry mass, and density, which determine LSM, SLA, density, and IE values (Gratani et al., 2018). Changes within the wet and dry seasons may also be dependent on variables such as light exposure, the height of the canopy, and gaps in the canopy itself (Smith et al., 2019). Leaves with thicker blades may accumulate more water in the dry season and may affect parameters such as LSM, LSA, and leaf density (Tian et al., 2016). In the degraded area, it was observed that trees were scarce, thus creating wide gaps in the canopy and exposing trees to extreme light. This may have an effect on the leaves' ability to retain water and photosynthesize effectively. Trees remained mesophyllous in both seasons for all study areas, which serves as an indication that although some parameters in locations were affected significantly by seasonality, small, insignificant changes were observed in their IE values.

#### 4.2.3 Leaf mass

Despite being an evergreen species, mangrove forests have a distinct seasonal greenness that is negatively related to litterfall and frequently lagged after the highest rainfall, with maximum greenness advancing in areas that receive more precipitation (Pastor‐Guzman et al., 2018). During the dry periods, mangrove forests, such as those found in the natural and restored areas, may transition to a water‐conserving semiarid environment, whereas during the rainy seasons, they can become well‐watered broadleaved deciduous forests. Such patterns could be caused by vapor pressure deficiencies, salt levels, or seasonal changes in air temperatures. Under these conditions, evapotranspiration levels are lowered, which lowers canopy conductance (Barr et al., 2014). During periods of rain and drought, photosynthetic productivity for mangrove species like *A. germinans* has been observed to drastically decline, whereas leaf fall has been reported to rise during intense dry seasons in mangrove locations (Loría‐Naranjo et al., 2014). Since this species is susceptible to changes in both canopy coverage as well as photosynthetic capacity, fluctuations in their leaves may be due to seasonal variations (Yaney‐Keller et al., 2019).

### 4.3 Correlation, regression, and PCA of A. germinans leaf traits

Our study revealed an increase in the number of positive associations during the wet season for the natural and restored mangrove ecosystems only. Parameters such as LL, LW, DW, TW, FW, AREA, and Peri maintained strong, positive correlations in all ecosystems. However, parameters such as LSM, LSA, density, and RWC consistently held strong, negative correlations in both seasons for all locations. Shi et al. (2019) noted that the leaf area, an essential functional characteristic of leaves, is correlated to leaf width and length and can vary depending on leaf morphology. As such, any changes to the leaf length or width can affect the leaf area, which consequently can affect other parameters such as LSA, Density, and RWC. The photosynthetic capabilities of plants are closely related to many leaf dimensions and scaling interrelations, including AREA, LSA, LSM, and thickness (Shi, Li, Niinemets et al., 2020). Although increasing the area of thicker leaves requires more fixed carbon than expanding the area of thinner leaves, the expansion in leaf area for a given increase in leaf mass may differ with the LSA. Hence, the increase in leaf area is closely related to LSA (Weraduwage et al., 2015). However, LSA shares an inverse relationship with LSM, which can explain the strong negative correlation values reported for all mangrove locations in both seasons (de la Riva et al., 2016). The results of the cormatrix analysis corresponded closely to those of the loading plots PC1 and PC2 for the PCA conducted in both seasons. These principal components were similar to those highlighted by Peel et al. (2017), who suggested that such morphological differences observed between leaf parameters may be attributed to shifts due to environmentally induced plasticity. Repeated multiple regression analyses on the relationship between ecosystem type and leaf parameters provided further substantial differences, which supports the strong, positive correlations identified within the study and shows the relationship that exists between ecosystem type and its influence on the *A. germinans* leaf parameters.

### 4.4 Leaf chlorophyll content of *A. germinans*

#### 4.4.1 Differences in *A. germinans* leaf chlorophyll content according to ecosystem type

Mangrove pigments have profound impacts on photosynthetic responses, stress resistance, and defensive systems. The predominant and frequent pigments in mangrove leaves are chlorophyll a and b (Croft & Chen, 2017). Moreover, according to Flores‐de‐Santiago et al. (2016), mangroves, such as *A. germinans*, are reported to possess chlorophyll a and b, with quantities varying based on the season and the mangroves' physiological state since freshwater availability and proximity influence the chlorophyll content of mangrove leaves. In certain cases, the amount of chlorophyll is closely correlated with the rate and intensity of photosynthesis in the vegetation (Dou et al., 2018). The spectral response of the photosynthetically active section of the leaf is marked by a low reflectance in the visible area and a high reflectance in the near‐infrared area, which is connected to the leaf shape (Baslam et al., 2020). In this study, the degraded mangrove ecosystem possessed the highest chlorophyll a and b, as well as the total overall chlorophyll content, followed by the natural ecosystem, and then the restored ecosystem. Significant results with respect to ecosystem type were seen only in the chlorophyll b and total chlorophyll contents. The results of our study coincided with those of Castellanos‐Basto et al. (2021), who concluded that the amount of chlorophyll in mangrove leaves varies depending on the species as well as the mangrove forest itself. Stressed mangroves, which are those that are vulnerable to anthropogenic or natural perturbations, have lower levels of incident light absorption, leaf chlorophyll, and general production (Heenkenda et al., 2015). However, contrary to popular belief, the chlorophyll content in the degraded area exceeded the values reported for the natural and restored areas. Additionally, Pastor‐Guzman et al. (2018) also noted that variations in the chlorophyll concentration of *A. germinans* may also be related to their DBH, basal area, and the maturation of their leaf tissue in accordance with the species, the existence of local features, seasons, and adjacent water sources Zulfa et al. (2020) highlighted that one of the most significant components in chlorophyll variations seems to be the reflectivity of the leaf surface, which can vary according to species. Flores‐de‐Santiago et al. (2012, 2013) observed increased chlorophyll a content in *A. germinans* found in deteriorated stands during the monsoon season, indicating that patterns of precipitation may have an impact on the biochemical components of leaves and, to some extent, the productiveness of the mangrove ecosystem. In mangrove forests, photoinhibition and other leaf‐damaging effects are more likely to occur when there is an increased frequency of light saturation (Comparini et al., 2020). Thus, the physiological stress caused by inundating periods, the changes in solar intensity, available water, interstitial salinity, climate impact, and moisture content is affiliated with the quantity of photosynthetic pigments in the leaf, which affects the leaf's development concurrently over time (Flores‐de‐Santiago et al., 2016).

#### 4.4.2 Differences in *A. germinans* leaf chlorophyll content according to seasonality

Our study reported considerable seasonal variations in the amounts of chlorophyll a, chlorophyll b, and total chlorophyll in the leaves of the mangrove ecosystems, notably in the natural and degraded sites. This was consistent with Flores‐de‐Santiago et al. (2016), who demonstrated that stressed mangroves exhibited a substantially larger fluctuation in chlorophyll concentration during the dry season as compared to mangroves during wet periods. Hence, it is probable that in light‐saturated conditions in stressed mangrove groups, photosynthesis may occur often which may result in photoinhibition and other negative impacts. Over the course of a year, the morphological traits and greenery of mangrove leaves change. In general, mangroves have high amounts of carotenes and chlorophyll in the dry season, although anthocyanin levels are highest during the rainy season (Panda et al., 2017). The biogeochemical dynamics of mangroves may also be impacted by changes in the hydrological regime which result in fluctuations of chlorophyll present throughout the year, with the wet season reporting particularly low levels (Kumar et al., 2017; Pérez et al., 2017). No significant relationships connecting leaf chlorophyll concentration and ecosystem type were observed in this investigation since in both seasons, the natural and restored mangrove ecosystems showed extremely weak linear relationships, according to the multiple regression analysis. However, a substantially linear relationship was seen in the degraded ecosystem during both rainy and dry seasons, which suggests that the amount of chlorophyll in leaves may be closely tied to that specific ecosystem type.

## 5 CONCLUSION

Our research revealed that *A. germinans* leaves, to some extent, exhibited variations in their traits according to habitat type and seasonality, confirming our hypothesis. The restored ecosystem exhibited significant variations in leaf measures, particularly in terms of length, width, perimeter, area, thickness, specific leaf area, mass (wet, dry, and turgid), and relative water content, in comparison to the other two ecosystems across both seasons. Moreover, it was observed that there were strong positive correlations in the three ecosystem types between leaf characteristics, including relative water content, mass, length, width, perimeter, and area, over both seasons. Conversely, there were substantial negative correlations between leaf‐specific mass, leaf‐specific area, density, and area. The results of our study also indicated that significant differences in leaf chlorophyll content were observed among various ecosystem types as well as seasonally. Specifically, the degraded ecosystem displayed the most prominent fluctuations in chlorophyll a, b, and total chlorophyll concentrations when compared to the natural and restored ecosystems. The findings of our study lend support to the notion that mangrove vegetation demonstrates a certain degree of plasticity in their plant structures to withstand substantial environmental changes and stresses, serving as a means of ongoing adaptation to these conditions and ensuring their survival.

## AUTHOR CONTRIBUTIONS

The first author conceptualized and designed the layout of the study. The first, second, and third authors aided in the acquisition, analysis, and interpretation of data, drafting and critically reviewing the article, and gave final approval of this version to be published.

## FUNDING INFORMATION

Our study was funded by donations provided by the Mid‐Atlantic Oil and Gas Company.

## CONFLICT OF INTEREST STATEMENT

The authors certify that this submission is original work and is not under review at any other publication. The authors have no potential conflicts of interest to declare.

## DATA AVAILABILTY STATEMENT

Datasets for this study are available through the Harvard Data Repository (Dookie, 2023).
